# Orchestration of antiviral responses within the infected central nervous system

**DOI:** 10.1038/s41423-024-01181-7

**Published:** 2024-07-12

**Authors:** Andreas Pavlou, Felix Mulenge, Olivia Luise Gern, Lena Mareike Busker, Elisabeth Greimel, Inken Waltl, Ulrich Kalinke

**Affiliations:** 1 Institute for Experimental Infection Research, TWINCORE, Centre for Experimental and Clinical Infection Research, a joint venture between the Helmholtz Centre for Infection Research and the Hannover Medical School, 30625 Hannover, Germany; 2 Department of Pathology, University of Veterinary Medicine Hannover, Foundation, 30559 Hannover, Germany; 3 Cluster of Excellence RESIST (EXC 2155), Hannover Medical School, 30625 Hannover, Germany

**Keywords:** Virus infection, Virus control within the brain, T cell recruitment to the brain, Microglia activation, Adaptive immunity, Infection, Innate immunity

## Abstract

Many newly emerging and re-emerging viruses have neuroinvasive potential, underscoring viral encephalitis as a global research priority. Upon entry of the virus into the CNS, severe neurological life-threatening conditions may manifest that are associated with high morbidity and mortality. The currently available therapeutic arsenal against viral encephalitis is rather limited, emphasizing the need to better understand the conditions of local antiviral immunity within the infected CNS. In this review, we discuss new insights into the pathophysiology of viral encephalitis, with a focus on myeloid cells and CD8^+^ T cells, which critically contribute to protection against viral CNS infection. By illuminating the prerequisites of myeloid and T cell activation, discussing new discoveries regarding their transcriptional signatures, and dissecting the mechanisms of their recruitment to sites of viral replication within the CNS, we aim to further delineate the complexity of antiviral responses within the infected CNS. Moreover, we summarize the current knowledge in the field of virus infection and neurodegeneration and discuss the potential links of some neurotropic viruses with certain pathological hallmarks observed in neurodegeneration.

## Viral encephalitis: broad spectrum of viruses with neurotropic potential and different disease outcomes

Viral encephalitis is a severe neuropathological condition that is associated with the presence of certain viruses within the central nervous system (CNS) that trigger inflammatory responses [1]. Although the syndrome is rare, severe encephalitis can be life-threatening and may result in serious consequences [1]. Clinically, viral encephalitis comprises multiple symptoms, ranging from mild, flu-like manifestations at the initial stage of the disease to severe, neurological impairments in advanced stages, including seizures, movement disorders, altered consciousness and even paralysis requiring immediate hospitalization [1]. Upon pathogen clearance, a significant number of patients present with long-term neurological sequelae that may also involve persistent cognitive dysfunctions and psychiatric deficits, which can drastically affect the patient´s daily routines [1–3]. Herpes simplex virus type 1 and 2 (HSV-1 and HSV-2, respectively) and varicella zoster virus (VZV) are the most common pathogens that can cause sporadic cases of encephalitis [1]. Herpes simplex encephalitis (HSE) has an annual incidence of approximately 1/250,000 worldwide [1]. During HSE, inflammatory lesions are usually observed within the mesiotemporal and orbitofrontal lobes together with the insular cortex, and affected individuals are in need of immediate acyclovir treatment [1]. Rabies virus (RABV), West Nile virus (WNV), Japanese encephalitis virus (JEV) and Dengue virus (DENV) are zoonotic pathogens that can cause endemic cases of viral encephalitis [1]. Patients infected with RABV develop severe clinical manifestations such as hypersalivation, hydrophobia, and agitation before reaching the paralytic form of the disease, which eventually results in coma [4]. WNV, JEV and DENV are mosquito-borne diseases that, depending on the immune status of the affected individual, may be self-limiting and asymptomatic or present with severe neurological manifestations such as headache, disorientation and seizures upon virus entry into the CNS [5–7]. Other important viruses that can cause severe encephalitis include La Crosse virus (LACV), Nipah virus (NiV), influenza A virus (IAV), eastern equine encephalitis virus (EEEV) and Chikungunya virus [1]. Since viral encephalitis is a severe disease characterized by vast complexity, the majority of the available literature discussed in this review describes experiments with flaviviruses such as WNV and JEV, herpesviruses such as HSV-1, rhabdoviruses such as vesicular stomatitis virus (VSV), and the arenavirus lymphocytic choriomeningitis virus (LCMV). Depending on the research question, each of the above infection models can help to delineate certain aspects of the disease, while not necessarily similar mechanisms apply to all viral encephalitis scenarios that are induced by different virus species and strains. In the mouse system, VSV and LCMV are two of the best-characterized pathogens and have been used for decades to establish the foundation of current knowledge in viral immunology.

## Viral CNS invasion strategies

Neurotropic viruses exploit various evasion strategies to bypass the cutaneous, mucosal and brain immune barriers and enter the CNS. The entry mechanisms of neurotropic viruses were extensively investigated by Cain et al. [8]. Briefly, Trojan horse-mediated CNS entry has been proposed to be a potential entry mechanism for WNV and NiV, which is further supported by the fact that both viruses can infect recirculating leukocytes [8]. Experiments using mice depleted of neutrophils or mice deficient in leukocyte adhesion molecules showed increased survival upon WNV infection, suggesting that the Trojan horse mechanism is indeed relevant for this virus [9, 10]. Moreover, infection of peripheral nerves and subsequent retrograde virus transport along axons into the CNS have been identified as a relevant mechanism in HSV-1, VZV and RABV infection, as reviewed by Taylor et al. and others [11]. Interestingly, disruption of the blood–brain barrier by viral proteases may lead to passive diffusion of infected cells or of intact virus particles into the CNS, which is an entry mechanism often observed during infection with flaviviruses such as WNV, JEV, and Zika virus (ZIKV) [8]. Furthermore, direct infection of brain endothelial cells may be a relevant CNS entry strategy, as shown in type I interferon receptor (IFNAR)-deficient mice that were infected with ZIKV [12]. In vitro experiments have characterized transcytosis as a potential invasion strategy for WNV using replication-deficient WNV-like particles [13]. Finally, nasal barriers can be manipulated by certain HSV-1 strains, neuroinvasive IAV, and potentially also by severe acute respiratory syndrome coronavirus 2 (SARS-CoV-2) to enter the CNS and cause long-lasting neurological sequelae [14–16].

## Nasal neuroinvasiveness as a paradigm of viral encephalitis

The olfactory epithelium contains olfactory sensory neurons (OSNs) that project their axons to single glomeruli within the glomerular layer of the olfactory bulb (OB) [15]. OSNs encounter a variety of viral pathogens daily, suggesting that several layers of immune protection must be in place to protect the CNS from viral infection via the olfactory route [15]. When viruses with neurotropic potential infect OSNs, a productive infection may occur, and virus particles reach the OB by retrograde axonal transport [15]. More specifically, as has been shown in experiments with pseudorabies virus (PRV), virus particles that enter the terminal axonal region of neurons induce the synthesis of host trafficking proteins that are required for efficient axonal transport of the virus [17].

**Fig. 1:**
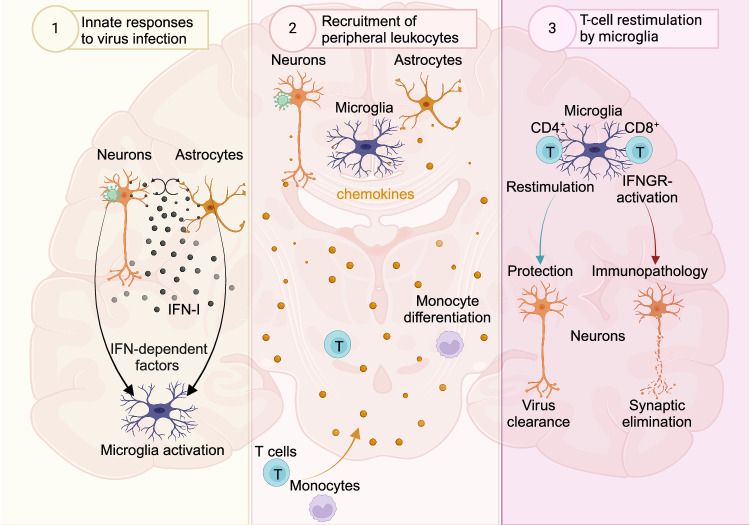
Schematic depiction of the sequence of immunological events triggered upon CNS virus infection. **(1)** Upon virus entry into the CNS, IFN-I signaling is essential for restricting virus propagation and promoting host survival. Astrocytes are important IFN-I producers, and together with neurons, they regulate the activation of microglia in an IFNAR1-independent manner [24, 25]. Microglial activation and recruitment to sites of infection are essential for virus control within the infected CNS [27, 29]. **(2)** Productive virus replication is established mainly within neuronal cells, leading to the induction of a potent chemokine response, which is tightly regulated by MyD88 signaling in a neuron-specific manner [30, 138]. Neuronal chemokine responses drive T-cell and monocytic cell recruitment to the infected CNS, which critically affects the outcome of the infection [30]. **(3)** At sites of infection, microglia are activated and proliferate, and they cross-present antigens to antigen-specific T cells within the infected CNS [29]. Infiltrated antigen-specific T cells are locally relicensed by microglia to exhibit optimal cytolytic activity that causes minimal tissue damage [29, 152]. However, under certain conditions, T-cell restimulation by microglia can lead to elimination of synapses and cognitive decline upon viral clearance from the CNS [120, 179]

Compared with mature OSNs, immature OSNs exhibit increased expression of low-density lipoprotein receptor class A domain-containing 3 (LDLRAD3), which is the main entry receptor of Venezuelan equine encephalitis virus (VEEV). Correspondingly, immature OSNs are more prone to VEEV infection than mature OSNs [18, 19]. Interestingly, LDLR was also shown to be the main entry receptor of VSV [20]. For HSV-1 the role of the olfactory entry route has been unclear for some time. Upon intranasal HSV-1 instillation of mice, robust infection of the olfactory epithelium and trigeminal ganglia was detected, whereas no productive replication of the virus was detected either within the OB or in the CNS parenchyma of wild-type (WT) mice [21]. However, deletion of Toll-interleukin-1 receptor (TIR) domain-containing adapter protein-inducing interferon beta (TRIF) and mitochondrial antiviral signaling protein (MAVS), which inactivates Toll-like receptor (TLR) 3 (TLR3) and RIG-I-like receptor (RLR) signaling, respectively, renders mice more susceptible to HSV-1 infection, and infected mice exhibit a phenotype resembling in many aspects that of HSE patients with inborn errors in the TLR3 axis [22]. Moreover, experiments in an HSV-1 eye scarification model using reporter mice and a Cre-expressing virus strain revealed that the virus replicated in the OB [23]. Once the virus reaches the CNS, innate immune responses of tissue-resident cells such as astrocytes are initiated, which are known to be abortively infected by HSV-1 and to mount robust type I interferon (IFN-I) responses [24, 25] (Fig. 1). Local IFN-I responses within the infected CNS have been shown to be protective, especially for neurons and astrocytes, since cell type-selective IFN alpha/beta receptor subunit 1 (IFNAR1) deletion renders mice susceptible to intranasal VSV instillation [26, 27]. Upon VSV infection, IFNAR1 stimulation of neurons and astrocytes regulates microglial activation, which is accompanied by a change in microglial morphology [27, 28]. During nasal infection, microglia are recruited to the site of infection, clonally expand and cross-present antigens to infiltrating antigen-specific T cells, this way coordinating the local adaptive immune response and potentially limiting tissue damage [27, 29] (Fig. 1). The infiltration of immune cells upon intranasal virus challenge is controlled by TLR signaling and, more specifically, by the adapter molecule myeloid differentiation primary response 88 (MyD88), which regulates chemokine expression in infected neurons [30] (Fig. 1).

The ability of the nasal barrier to restrict viral neuroinvasion became relevant during the coronavirus disease 2019 (COVID-19) pandemic, even though SARS-CoV-2 is mainly a respiratory pathogen. In the initial stage of the pandemic, anosmia and loss of taste were considered typical signs of SARS-CoV-2 infection [31]. Histological examinations of the olfactory mucosa revealed few cases in which OSNs were infected by SARS-CoV-2, suggesting that under certain conditions, the nasal neuroepithelium could be a potential route of virus entry into the CNS [32]. However, in mice, intranasal administration of SARS-CoV-2 resulted in olfactory dysfunction due to the disruption of olfactory cilia, in which the olfactory receptors are located, and not due to direct infection of OSNs [33, 34]. Interestingly, even upon intranasal instillation of SARS-CoV-2, viral neuroinvasiveness is not observed, and microglia within the OB and hippocampus of hamsters and mice are activated and express IL-1β, which has been shown to negatively affect the neurogenic potential of neuronal precursors [35, 36]. Moreover, OSN function is essential for the survival of periglomerular dopaminergic neurons within the OB. Upon SARS-CoV-2 challenge in mice, a reduction in the number of tyrosine hydroxylase-expressing neurons is observed in the OB, suggesting that the infection has at least a transient effect within the neuronal compartment of the OB [34]. Whether repeated SARS-CoV-2 infections affects the neuronal compartment of the OB in the long-term remains to be elucidated. Due to the accumulating evidence on long-term neurological sequelae in individuals infected with SARS-CoV-2, delineating whether the nasal barrier plays a role in regulating CNS-resident cellular responses upon SARS-CoV-2 infection is mandatory [37].

## Deciphering genetic predispositions in patients with viral encephalitis illuminates relevant in vivo mechanisms for virus control within the CNS

**Fig. 2:**
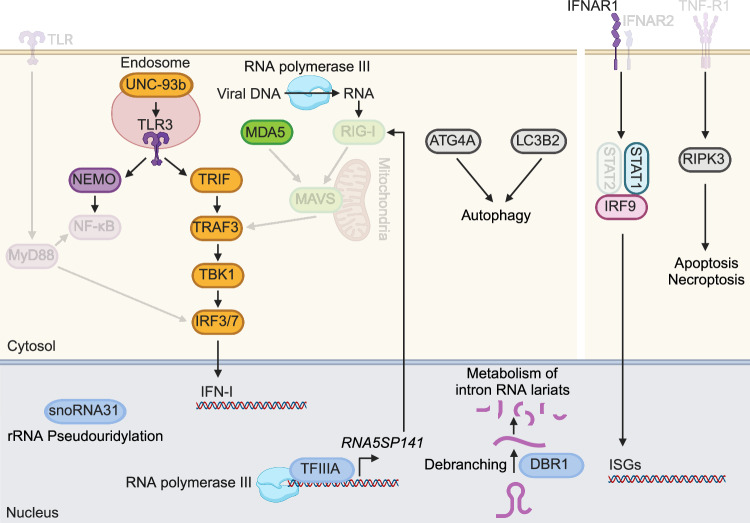
Detrimental inborn errors in virus sensing and IFN-I signaling illuminate relevant mechanisms of protection against viral encephalitis. Inborn errors of components of sensing pathways and IFN signaling important for protective innate immune responses during virus infection in the CNS that are described in this review are highlighted in bright colors. Mutations in the TLR3 gene were found in patients who presented with HSE [49] and VZV encephalitis [57], enterovirus rhombencephalitis [56], and influenza A virus-associated encephalitis [58]. Additionally, a TLR3 mutation was proposed as a TBE risk factor [59]. TLR3 is therefore critical for protective responses during viral encephalitis of multiple viral etiologies. TLR3 and other TLRs are trafficked and stabilized by UNC-93b [45, 47, 48]. UNC-93b deficiency has been detected in HSE patients [50]. Furthermore, HSE is associated with mutations in the TLR3 adapter molecule TRIF [54] and in the signaling molecules TRAF3 [53], TBK1 [52], IRF3 [51], and IRF7 [55]. A mutation in the RNA sensor MDA5 was identified in a child with EV71 rhombencephalitis [56]. Host rRNAs can trigger RIG-I activation [70]. TFIIA (*GTF3A*), a transcription factor for the RNA polymerase III complex, induces the transcription of rRNA, while SnoRNA31 (*SNORA31* locus) is responsible for the pseudouridylation of rRNA. HSE is associated with mutations in both *GTF3A* [72] and *SNORA31* [75], while missense mutations in RNA polymerase III are associated with severe VZV infections, including encephalitis [64]. Defective genes related to apoptosis and necrosis, such as *RIPK3* [78], as well as genes connected to autophagy, including *ATG4* and *MAP1LC3B2* [79], have been shown to be associated with HSE. Several inborn errors in genes related to IFNAR signaling, including *IFNAR1* [39], *STAT1* [38], and *IRF9* [40], are associated with HSE. *IRF9* deficiency is further associated with multiple viral infections, including DENV and ZIKV [40]

The identification of single inborn errors in individuals who suffered from viral encephalitis, along with the monitoring of clinical parameters, helped to delineate the pathomechanisms of the disease. First, signal transducer and activator of transcription 1 (*STAT1*) inborn errors were reported in two infants who succumbed to a lethal viral disease together with mycobacterial dissemination, but the viral etiology of the second infant remained unclear [38]. The first infant succumbed to herpes meningoencephalitis after discontinuation of acyclovir treatment. This child had a homozygous two-nucleotide deletion within exon 20 of *STAT1*, which generated a premature stop codon that resulted in a truncated form of STAT1 lacking the Src homology 2 (SH2), tail and transactivation domains [38]. Although the role of type II interferon (IFN-II) in the pathology of this individual could not be determined, this was the first reported case highlighting IFN-I responses as an essential protective mechanism against viral encephalitis. Later, two children with HSE were identified who carried a large deletion within the *IFNAR1* locus, which does not directly impair IFNAR1 expression on the cell surface but results in a truncated intracellular domain that is unable to promote signaling [39]. Furthermore, interferon regulatory factor 9 (*IRF9*) deficiency was identified in a family suffering from multiple infections, with one miscarriage associated with DENV and ZIKV coinfection and one child presenting with HSV-1 meningoencephalitis [40] (Fig. 2). During the SARS-CoV-2 pandemic, the discovery that autoantibodies neutralizing IFN-I responses could be involved in causing enhanced disease severity paved the way for the evaluation of the role of autoantibodies in several other viral diseases [41]. Neutralizing autoantibodies against IFN-I were detected in more than 40% of severely WNV-affected individuals with life-threatening neurological sequelae such as encephalitis, meningitis, and paralysis [42]. The reports from the inborn errors summarized above, together with the finding that an individual who presented with severe WNV encephalitis recovered upon subcutaneous IFN-α2 treatment, suggest that IFN-I activation is essential in both the CNS and peripheral compartments to prevent the development and progression of viral encephalitis [43]. Interestingly, the WNV patient was reported to be a *GATA2* heterozygous single-base deletion carrier. *GATA2* deficiency is known to cause a wide range of clinical phenotypes, including severe infections.

At the virus-sensing level, impaired TLR3 signaling has been reported to play a central role in the development of viral encephalitis, especially in HSE after primary infection. Although HSV-1 is a DNA virus, during HSV-1 infection, dsRNA intermediates are formed that can induce TLR3-mediated responses under certain conditions [44]. In an N-ethyl N-nitrosourea mutagenesis screen in C57BL/6 mice, mutations were detected that rendered murine macrophages nonresponsive to certain TLR ligands that are sensed by endolysosomal TLRs [45]. At the same time, similar observations were made with peripheral blood mononuclear cells (PBMCs) and fibroblasts from two individuals who developed HSE [46]. These observations resulted in the identification of two distinct mutations of the *UNC93B1* gene, which nowadays is known to control the endolysosomal trafficking and stability of TLR3, 7, and 9 [45, 47, 48]. Later, the identification of a heterozygous mutation within the predicted dsRNA binding cleft of TLR3 discovered in two unrelated children with HSE, together with HSV-1 infection experiments in neuronal cells reprogrammed from induced pluripotent stem cells (iPSCs) generated from these HSE patients, highlighted that TLR3 signaling is essential within the CNS compartment upon HSV-1 encounter [49, 50] (Fig. 2).

More recently, mutations in components that are relevant only for TLR3-mediated signaling (TLR3 specific: *TRIF*) and that are relevant for signaling by TLR3 and other sensors (TLR3 nonspecific: *TRAF3, TBK1, IRF3*, and *IRF7*) have been identified in several HSE-affected individuals, clearly indicating that TLR3 signaling, especially during childhood, is of key relevance for preventing HSE [51–55] (Fig. 2). Furthermore, these data provide evidence for the in vivo relevance of TLR3 for sensing HSV-1. Interestingly, compound heterozygous TLR3 deficiency was identified in a child who developed viral rhombencephalitis caused by a positive-sense single-stranded RNA enterovirus (EV), echovirus 30 (EV30), suggesting that in the case of RNA virus infection, intact TLR3 signaling is also required to prevent encephalitis [56]. Moreover, TLR3 missense mutations and rare genetic variants of TLR3 that have not yet been functionally characterized were recently reported in patients with VZV encephalitis, influenza-associated encephalopathy, and tick-borne encephalitis virus (TBEV) meningoencephalitis. However, more functional assays are needed to delineate the underlying mechanisms [57–59].

Nuclear factor kappa-light-chain-enhancer of activated B cells (NF-κB) is a central signaling component that triggers the transcriptional initiation of several proinflammatory genes [60]. Patients with deficiencies or mutations in its activator NF-κB essential modulator (NEMO) present with severe immunodeficiency and are highly susceptible to several infectious diseases, including HSE [61, 62]. RNA polymerase III transcribes nuclear DNA to ribosomal RNA (rRNA), transfer RNA (tRNA), and other small RNA species. However, RNA polymerase III additionally converts cytosolic viral DNA into RNA, which is sensed by RIG-I [63] (Fig. 2). The importance of RNA polymerase III during DNA virus infection of the CNS was highlighted by RNA polymerase III mutations identified in children with severe VZV encephalitis whose PBMCs showed diminished IFN-I responses upon DNA stimulation [64]. Finally, two individuals who suffered from HSE were identified to carry nonsynonymous deletion variants in *MASP2*, which is part of the lectin pathway and controls the cleavage of the complement components C2 and C4 [65].

Several animal studies highlighted the significance of RIG-I-like receptors (RLRs) as the main sensing platform for RNA virus infections in the CNS compartment [66–69]. The recent identification of a homozygous mutant of *IFIH1*, which encodes melanoma differentiation-associated protein 5 (MDA5), in a child with enterovirus 71 (EV71) rhombencephalitis further emphasized the significance of this sensing pathway in human disease [56]. Although RLR signaling is of key importance for the sensing of RNA viruses, reports have shown that RLR signaling is also activated during DNA virus sensing [70]. Interestingly, during HSV-1 infection, several host-derived noncoding RNAs bind to RIG-I, with the top hits being 5S ribosomal RNA pseudogenes such as *RNA5SP141* [71]. These ribosomal pseudogenes usually bind to proteins and thus coordinate intrinsic cell functions. However, several viruses that have sophisticated evasion and transcriptional shutoff strategies, such as herpesviruses, can downregulate the expression of certain proteins that are no longer bound by ribosomal pseudogenes, thus allowing pseudogenes to bind to RIG-I, which results in enhanced RIG-I activation and increased IFN-I responses [71]. Similarly, a child who presented with HSE was identified to carry compound heterozygous mutations in *GTF3A*, which encodes the transcription factor TFIIIA that regulates the transcription of ribosomal RNAs [72, 73]. The identified mutations led to impaired binding of TFIIIA to DNA, which resulted in the diminished initiation of transcription of several noncoding RNAs, including *RNA5SP141*. The inability to transcribe such pseudogenes leads to reduced RIG-I activation and consequently to impaired protection against HSV-1 infection [72]. These *GTF3A*-deficient patients presented with a common variable immunodeficiency (CVID) phenotype, which has been shown in several cases to be associated with HSE [72, 74]. Furthermore, five patients with HSE were identified to have deleterious mutations in the SNORA31 locus, which is a gRNA regulating chemical modifications, termed pseudouridylation. SNORA31 mutations impair the pseudouridylation of the ribosomal 18S RNA U218, which affects the sensitivity of cortical neurons to HSV-1 infection, but the precise mechanism is not yet clear [75]. Patients with HSE associated with primary inborn genetic errors primarily presented with forebrain HSE. Brainstem viral encephalitis is rare and was recently identified in several patients carrying mutations in *DBR1*, which encodes an RNA lariat-debranching enzyme. The accumulation of intronic RNAs may be toxic to cells, thus rendering the brainstem neuronal compartment more susceptible to viral infection and replication. These patients suffer from brainstem encephalitis due to HSV-1, influenza B virus, or norovirus infection [76] (Fig. 2).

The mechanisms of cell death and autophagy are important biological functions, especially during infection, and can eventually affect the outcome of infection by regulating the magnitude of inflammatory responses or the spatiotemporal kinetics of viral replication and propagation. A histopathological analysis revealed that many cells in HSE lesions undergo apoptosis [77]. Similarly, a patient suffering from HSE was recently found to carry compound heterozygous mutations in *RIPK3*, which regulates cell-mediated death via apoptosis and necroptosis [78]. Furthermore, deleterious mutations in two autophagy genes, *ATG4* and *MAP1LC3B2*, were identified in two patients who suffered from HSV-2 recurrent lymphocytic meningitis [79] (Fig. 2). Overall, the identification of genetic variants associated with viral encephalitis has tremendously expanded the knowledge about relevant pathways in viral encephalitis. However, the molecular mechanism of most viral encephalitis cases is still unknown, and additional studies are needed to elucidate the factors that are relevant for the control of viral infection within the CNS.

## Modeling brain diseases using neuroimmune organoids

Obtaining non-fixed human brain tissue is still challenging, which has impeded progress toward elucidating brain pathologies associated with viral infection. However, the advent of human iPSC technology has facilitated the development of 3D brain organoids that can be used to model neurotropic viral diseases [80]. Brain organoids recapitulate the intricate structure and diverse functions of the human brain [81], overcoming limitations associated with conventional 2D cell culture systems [82]. Unlike classical organoids, the development of neural organoids supplemented with microglia, i.e., immunized organoids, has allowed the exploration and manipulation of immune responses within organoids. Using immunized organoids, Samudyata et al. [83] observed microglia-mediated synaptic pruning following viral infection, which mirrored phenotypes documented in neurodegenerative disorders. Infection studies using brain organoid models reported ZIKV replication within neural precursor cells, astrocytes, and neurons, resulting in structural defects and cell death, which are key features associated with ZIKV-induced microcephaly [84–86]. Indeed, ZIKV infection in immunized organoids leads to microglial activation and the subsequent induction of proinflammatory cytokines such as IL-6, IL-1β, and tumor necrosis factor (TNF), thus linking microglia with ZIKV-induced neuropathology [87]. Using human cytomegalovirus (HCMV), which is another virus associated with microcephaly, Sun et al. [88] observed a disruption of organoid morphology, impaired neurogenesis and the formation of neural rosettes following infection.

Although neurological manifestations in COVID-19 patients have been reported [89], the neuropathology associated with SARS-CoV-2 infection remains elusive. Yi et al. [90] reported pronounced expression of ACE2 in dorsal forebrain organoids and increased susceptibility to SARS-CoV-2 pseudovirus infection. Using cortical, hippocampal, hypothalamic, and midbrain organoids, several groups have reported moderate SARS-CoV-2 infection of neurons [91] and astrocytes [92] and robust infection of choroid plexus epithelial cells [93]. Moreover, by utilizing forebrain and midbrain organoids, Hou et al. [94] showed an enhanced replication efficiency of variant Omicron BA.2 with productive viral infection within dopaminergic neurons, indicating that SARS-CoV-2 infection can undermine neural circuit integrity.

Although brain organoids have proven to be a suitable method for modeling certain aspects of viral brain infection in vitro, the system remains incapable of recapitulating all relevant aspects, including infiltration of the CNS with peripheral immune cells. Furthermore, if relevant components of virus control have been identified in vivo, dissecting whether the respective components play a role during virus invasion into the CNS or during virus control within the CNS will remain challenging.

## Microglial responses determine antiviral outcomes and the establishment of long-term neurological sequelae

Microglia are resident mononuclear phagocytes of the CNS that are relevant for tissue surveillance [95]. Microglia are activated by a variety of triggers, and their activation is essential for the control of viral infection in the CNS and thus critically determines disease outcomes [27, 96–102]. Microglia express a variety of pattern recognition receptors, including TLRs [103] and RLRs [104], and therefore readily respond to invading pathogens, including HSV-1 [104], and pathogen-associated patterns (PAMPs), such as the bacterial cell wall component lipopolysaccharides (LPS) [105] or double-stranded RNA [106], which arise during the replication of positive single-stranded RNA-encoded viruses such as WNV or TBEV.

Postmortem histological analyses of brain tissue from patients with viral encephalitis revealed the importance of microglia during the course of the disease, and microglia were found to be located in close proximity to T cells [101, 107]. The discovery of IL-34 as an alternative CSF1R ligand that is essential for microglial development and the increased sensitivity of IL-34-deficient mice to infection with attenuated WNV highlighted the role of microglia in protection against CNS infection [97]. Moreover, microglial depletion experiments have shown that mice are highly susceptible to various viral challenges [27, 98–100, 102, 108]. Upon virus entry into the CNS, microglia are activated, MHC I and MHC II molecules are upregulated, proliferation and clonal expansion occur, and microglia are recruited to sites of active viral replication [27, 29, 101, 109, 110]. Interestingly, in the PRV mouse model, researchers proposed that microglial recruitment to virus-infected brain areas is controlled by P2RY12, which senses ATP molecules released from virus-infected cells [101]. However, monocyte recruitment to infected areas has been shown to be P2RY12 independent [101]. Consequently, another purinergic receptor, P2RY13, was also detected to be expressed within the CNS parenchyma, and it was proposed to be an interferon-stimulated gene upon intranasal VSV instillation. This result suggested that purinergic receptors other than P2RY12 and 13 might play a role in CNS infection [111]. After HSV-1 infection, microglia mount IFN-I responses in a cGAS/STING-dependent manner [109]. However, cGAS can also limit IFN-I responses by instructing myeloid cells to undergo apoptosis and thus protect sensitive neuronal compartments [107]. Interestingly, in a mouse model in which a point mutation was introduced in the C-terminal tail of STING that is responsible for IFN-I-mediated responses, STING signaling was not essential for regulating IFN-I-mediated protection upon HSV-1 eye scarification infection but rather for regulation of autophagy [112].

Microglia are capable of phagocytosing and digesting invading viruses [113], but in some cases, they may also support intracellular virus replication [114]. Under homeostatic conditions, ramified, ameboid, and pseudopodic microglia are found in the CNS [115]. Ramified microglia exhibit long dendrites that enable efficient surveillance of the environment [116], while ameboid microglia have an oval-shaped cell body with a ruffled membrane and contain a larger nucleus [115]. Pseudopodic microglia have a mixed morphology with a ruffled membrane and few cytoplasmic projections termed pseudopodia that protrude from their cell body [115]. Upon activation, microglia typically retract their dendrites, acquire an ameboid or pseudopodic morphology, become more motile, and increase their phagocytic capacity [115, 117].

Microglial activation is critically needed to control viral infection of the CNS; however, pathological effects following microglial activation, which are mediated by neurotoxicity or synaptic elimination during acute inflammation or in the recovery phase after viral encephalitis, have been observed [118–121]. In fact, microglial inflammatory responses cause neuronal apoptosis following LPS stimulation in vitro [118] and after ZIKV infection in vivo [120]. Post-WNV infection, complement signaling [121] and IFNGR signaling [120] of microglia promote synaptic removal, which leads to cognitive deficits. Similarly, in the mouse viral déjà-vu model infected with LCMV, which closely recapitulates Rasmussen’s encephalitis, myeloid cells contribute to neuronal synapse removal, which leads to movement impairments [122]. Therefore, microglial activation is needed for the control of viral infections in the CNS, but microglia can also cause long-term neurological sequelae.

## Checkpoints of immune cell infiltration during CNS infection

Upon viral CNS entry, immune cells infiltrate the infected CNS parenchyma to control virus propagation and to restrict viral dissemination throughout the entire CNS. Infiltration of the infected CNS with immune cells from the periphery involves several critical steps. Initially, the drainage of viral antigens, or even of intact virus particles, to secondary lymphoid organs, especially the cervical lymph nodes, is a fundamental step in mounting protective immune responses upon CNS infection. Furthermore, chemokines produced by tissue-resident cells that respond to virus infection eventually define which immune cell types infiltrate the infected brain and to which anatomical localizations these cells will home. Finally, local relicensing of immune cells that infiltrate the CNS, such as antigen-specific CD8^+^ T cells, by tissue-resident cells is needed to coordinate and fine-tune the function and magnitude of the immune response within the infected CNS.

The significance of secondary lymphoid organs as highly organized lymphoid structures that are relevant for the control of LCMV and VSV infection was revealed by elegant experiments with alymphoplasia and *Hox11*-deficient mice [123]. These mice lack lymph nodes and spleens, respectively, and challenge with viruses that require T- and B-cell responses to control chronic or acute infection revealed that secondary lymphoid tissues are needed for effective T-cell priming and CNS protection [123]. The recharacterization of the lymphatic drainage system of the CNS compartment under steady-state conditions paved the way for the delineation of the role and responses of the lymphatic vessels during neuroinflammation [124–127]. CNS infection caused by a broad range of neurotropic pathogens leads to a reduced capacity of meningeal lymphatic vessels (MLVs) to drain antigens into deep cervical lymph nodes [128]. Moreover, Japanese encephalitis virus (JEV) and VSV CNS infections cause downregulation of the transcription factor PROX1, which is essential for lymphatic valve function and smooth muscle cell contraction in MLVs, thus leading to dysfunctional MLVs [128]. Pretreatment of mice with recombinant VEGF-C, which is known to promote an increase in the diameter of lymphatic vessels and the expansion of functional MLVs, significantly improved the survival of mice upon JEV challenge [127, 128]. These data clearly indicate that the lymphatic system is of pivotal significance and can be therapeutically exploited during viral encephalitis [126–128]. In experiments with VSV and vaccinia virus, sinus subcapsular macrophages are productively infected upon viral particle transport to the draining lymph node [129]. Then, naïve CD8^+^ T cells relocalize to the peripheral interfollicular area adjacent to the sinus subcapsular macrophage lining. Upon relocalization, T cells interact with infected or cross-presenting DCs to achieve optimal activation [129].

**Fig. 3:**
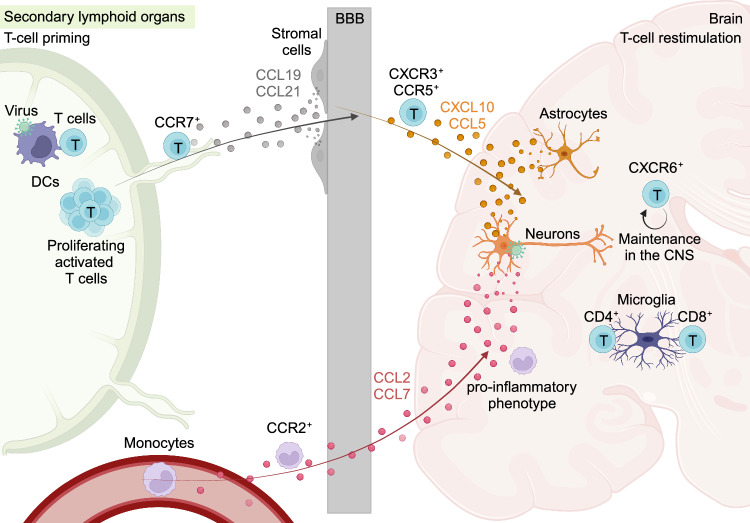
Chemokines derived from CNS-resident cells drive the recruitment of peripheral leukocytes into the infected brain. T cells are primed by DCs in secondary lymphoid organs in the periphery and proliferate [250]. Stromal cells (fibroblastic reticular cell-like cells surrounding the perivascular spaces and endothelial cells of the meningeal blood vessels) secrete CCL19 and CCL21, which recruit CCR7^+^ CD8^+^ T cells to the BBB. CXCL10 and CCL5 derived from neurons in the VSV model [30] or from astrocytes in the LCMV Traub model [143, 144] recruit T cells via CXCR3 and CCR5, respectively. CXCL10 is especially important for CXCR3^+^ CD8^+^ and gB^+^ CD8^+^ T cells [138, 139]. CXCR6 signaling leads to the maintenance of T cells in the CNS. Microglia interact with CD4^+^ and CD8^+^ T cells and activate previously primed antigen-specific T cells [29, 98, 250, 251]. Monocytes are recruited from the blood stream via the CCL2/CCL7–CCR2 axis [159], by CCL2 originating from neurons in the TMEV model [160]. CCR5 might contribute to leukocyte recruitment during WNV infection [145]

The establishment of a chemokine gradient within the infected CNS is necessary for the induction of immune cell infiltration, mainly CD8^+^ T cells, which has been shown to be essential for protection against neuroinvasive virus infection [23, 29, 30, 130–135]. The discovery of the measles virus (MV) receptor CD46 allowed the development of an MV-encephalitis mouse model using transgenic mice expressing human CD46 under the control of a neuronal promoter [136]. Intracerebral inoculation of MVs in these transgenic mice leads to predominant infection of neuronal cells that then express CXCL10 and CCL5, thus recruiting T cells to the infected CNS parenchyma [137]. However, in that study, the authors remained unclear on whether these chemokine responses were essential for protection. The definitive answer was obtained with CXCL10-deficient mice that showed increased sensitivity to WNV infection or by injecting CXCL10-blocking antibodies into infected mice [138]. Compared with WNV- or HSV-1-infected WT mice, CXCL10-deficient mice showed decreased infiltration of the CNS by CXCR3^+^ CD8^+^ T cells or gB^+^ CD8^+^ T cells, respectively [138, 139]. Similar sensitivity to WNV infection as detected in CXCL10-deficient mice was  also observed in CXCR3-deficient mice [140]. Viruses with highly sophisticated immune evasion programs, such as HSV-1, can downregulate CXCL9 expression via the viral kinase UL13, thus diminishing the infiltration of CD8^+^ T cells within the infected CNS [135]. The observations that MyD88-deficient mice are heavily susceptible to WNV and VSV brain infections and that they show reduced numbers of CNS-infiltrating leukocytes raised the question of whether infected neurons solely regulate the chemokine response via the adapter molecule MyD88 [141, 142]. By exploiting the RiboTag approach, i.e., cell-selective Cre-mediated ribosomal tagging and subsequent RNA sequencing of the translatome of selected CNS-resident cells, neurons were identified to be the major chemokine producers upon VSV infection of the CNS [30]. Moreover, the selective neuronal reconstitution of MyD88 signaling within the infected CNS phenocopied the survival of control mice and decreased the number of CNS-infiltrating leukocytes upon intranasal VSV infection [30] (Fig. 3). However, upon intracranial infection with the LCMV Traub strain, which causes CD8^+^ T-cell-mediated immunopathology and meningitis in immunocompetent mice, CXCL10 expression seems to originate mainly from astrocytes, and CXCL10 strongly drives the infiltration of CXCR3^+^ CD8^+^ T cells into the meningeal compartment [143, 144]. Notably, CCR5 signaling has also been shown to have a significant impact on the protection and infiltration of T cells within the CNS upon WNV, JEV, and LGTV infection [145–147].

When CD8^+^ T cells are primed and reach inflamed tissue, fibroblastic reticular-like cells surrounding the perivascular space together with the endothelial cells of the meningeal blood vessels express high levels of CCL21 and CCL19 to promote the migration of CCR7^+^ CD8^+^ T cells within the MHV-A59-infected CNS parenchyma [148]. CCR7 signaling in these CD8^+^ T cells facilitates their exit from the meningeal blood vessels but also drives their localization within the infected CNS [148]. CCR7 signaling-dependent blood vessel exit does not seem to be essential for the CNS migration of CD4^+^ T cells [148] (Fig. 3). In contrast, CXCL12-expressing endothelial cells retain CD8^+^ T cells within perivascular spaces upon WNV infection [149]. By antagonizing CXCR4-CXCL12 signaling, the release of CD8^+^ T cells to the infected parenchyma is increased, and therefore viral control is promoted [149]. The expression levels of CXCL12 are also regulated by IL-1R signaling, which has been shown to be critical for T-cell trafficking during neuroinfection [150].

Intraparenchymal antigen-specific T cells interact with microglia in a process that is essential for viral clearance from the CNS in models of acute or persistent viral infection [29, 151]. Local intraparenchymal T-cell restimulation from CNS-resident and infiltrating antigen-presenting cells can optimally coordinate the CD8^+^ T-cell response during viral encephalitis [152] (Fig. 3). Furthermore, intraparenchymal CD8^+^ T-cell cytolytic function is also regulated by the presence of CD4^+^ T cells upon infection with the neurotropic mouse hepatitis JHM strain and congenital murine cytomegalovirus (MCMV) in a manner that requires further investigation [153, 154].

Although T-cell recruitment is critical for the control of viral encephalitis, also myelomonocytic cells massively infiltrate the infected CNS parenchyma. Debate is ongoing since, thus far, only a few studies have analyzed their roles in viral encephalitis. The depletion of monocytes and macrophages by treatment with clodronate-loaded liposomes before WNV and VSV infection exacerbated the sensitivity of mice to lethal infection with high rates of viral neuroinvasiveness, highlighting the importance of myeloid cells at least during the acute phase of infection [141, 155, 156]. CCR2 is an important chemokine receptor that regulates the exit of monocytic cells from the bone marrow in mice [157]. CCR2-deficient animals are highly susceptible to WNV infection [158]. However, competitive repopulation experiments in which CCR2^−/−^ and CCR2^+/+^ monocytes were mixed at a 1:1 ratio and injected into CCR2-deficient recipient WNV-infected mice showed that the monocyte cell ratio was similar between the CNS and blood. Furthermore, the disappearance of CCR2^−/−^ monocytes from the blood even early after transfer suggested that CCR2 is only required for monocytes to recirculate from the bone marrow to the blood and is not needed for monocytes to infiltrate the infected CNS [158]. In the WNV mouse model, CCL2 and CCL7 are important ligands for CCR2 and affect monocyte infiltration to the CNS; however, only CCL7 deficiency is associated with increased sensitivity to infection [159]. In the TMEV model, hippocampal neurons are the predominant source of CCL2 upon infection, and neuron-specific genetic ablation leads to reduced infiltration of monocytes within the brain [160]. However, an intriguing study showed that CCR2 deficiency results in better clinical outcomes in mice upon JEV infection, while CCL2 deficiency renders mice highly susceptible to infection [161]. CCL2 deficiency led to increased accumulation of monocytes within the JEV-infected brain compartment, suggesting that monocytes may require other chemokines, and these results thus contribute to the discussion on whether excessive monocyte infiltration may cause pathogenic effects on the CNS parenchyma under certain conditions [161, 162] (Fig. 3). To this end, in the LCMV model, monocytes and neutrophils clearly contribute to CNS vascular injury, leading to Evans blue leakage from the meningeal blood vessels to the CNS parenchyma [163]. The depletion of neutrophils in CCR2-deficient mice prolonged the survival of the animals upon LCMV infection [163]. Monocyte recruitment patterns were shown to be affected during HSV-1 and TMEV infection [110, 164]. These data clearly suggested that other chemokine receptors, such as CCR5, may also be involved in the trafficking of monocytes from the periphery to the CNS [145].

## The protective effect of CD8^+^ T cells during viral encephalitis

CD8^+^ T cells play a crucial role during the course of virus infection and undergo several steps, from antigen-specific T cell priming in secondary lymphoid organs to T cell infiltration of the CNS and local T cell restimulation within the CNS, to efficiently restrict viral propagation within the CNS [165]. The majority of the acquired knowledge on the role of CD8^+^ T cells in viral encephalitis originates from mouse experiments with several model pathogens, including WNV, JEV, VSV, LCMV, Borna disease virus, and HSV-1 [29, 30, 130, 132, 134, 135, 166]. Upon reaching the infected area of the CNS parenchyma, CD8^+^ T cells produce molecules that are relevant for viral clearance. Perforin has been shown to be essential for CD8^+^ T-cell function as indicated by perforin-deficient mice being sensitive to WNV infection [167]. Interestingly, of the few perforin-deficient mice that survived the infection, the virus could be reisolated from the brain even at 35 days postinfection, suggesting that the ability of perforin to lyse the cell membrane of infected cells is important for virus control [167]. Furthermore, similar results were obtained upon WNV infection in gld mice, which contain a point mutation in *FasL*, and in TNF-related apoptosis-inducing ligand (TRAIL)-deficient mice, suggesting that other CD8^+^ T-cell-mediated effector functions are needed to optimally protect against WNV infection [168, 169]. However, during JEV infection, only IFN-γ seems to provide a significant survival advantage over other cytolytic effector pathways, such as the perforin, granzyme A/B and Fas-mediated death pathways [170, 171]. Notably, CD8^+^ T cells can clear infections within the CNS in a noncytopathic manner, as observed during VSV, Sindbis virus, and MHV brain infection, which is in contrast to what is usually observed in vitro [29, 172, 173]. T-cell-dependent cytokine responses, such as those involving IFN-γ, have been shown to play a central role in noncytolytic viral clearance within the CNS, although different neuronal subtypes may exhibit divergent responses to cytokine stimulation [172]. Interestingly, CD8^+^ T-cell-mediated lytic granules were shown to noncytolytically restrict HSV-1 reactivation by selectively targeting the viral life cycle without causing neuronal apoptosis [174].

The memory CD8^+^ T-cell compartment has been shown to play an important role in suppressing the reactivation of viruses and CNS entry for recurrent infections. More precisely, intracerebral infection with an attenuated LCMV strain leads to the generation of tissue-resident memory CD8^+^ T cells that seed-specific anatomical locations of the brain, such as the meninges and the choroid plexus, and undergo homeostatic proliferation, which is important for their maintenance within the brain [175]. Interestingly, during reinfection, these memory CD8^+^ T cells expand and provide faster viral clearance [175]. Peripheral infection and immunization can also generate brain-resident CD8^+^ T cells that seed these specific anatomical locations in the CNS [176]. Furthermore, brain-resident memory CD8^+^ T cells are also generated after congenital MCMV infection, and they are long-lived cells seeded in the CNS [177]. Only prolonged depletion strategies deplete the resistant memory CD8^+^ T-cell compartment in the brain, leading to reactivation of MCMV and subsequent detection of cells expressing immediate early genes, which suggests viral reactivation [177]. Similarly, the accumulation of memory antigen-specific T cells within the OB has been observed in an ocular HSV-1 infection model, even after 60 days post infection [23]. Such brain memory T cells can also be found in the human brain [178].

Nevertheless, the function of CD8^+^ T cells is essential during the acute phase of infection. However, CD8^+^ T cells may cause severe neuropathology with cognitive impairment. In a model of WNV-induced cognitive dysfunction, brain memory CD8^+^ T cells persist in the CNS by sensing microglia-derived CXCL16. CXCL16 targets CXCR6^+^ CD8^+^ T cells to promote their maintenance in the brain, eventually leading to increased IFN-γ production [179] (Fig. 3). Presynaptic elimination is mediated through microglia-specific IFNGR signaling, which can cause long-term cognitive impairment [120].

## From bulk to single-cell omics: tools to investigate microglial biology in health and disease

### Conventional bulk RNA sequencing

The development of technologies that allow analyses of cellular transcriptomes, such as quantitative PCR (qPCR) [180], microarrays [181, 182], NanoString [183] and RNA sequencing (RNA-Seq) [184], has been instrumental in the identification of core microglial signatures. Although qPCR, NanoString and microarrays have offered valuable insights into microglial biology, they have only allowed low-throughput assessments of the transcriptome. Ultimately, RNA-seq has become the most valuable technique for deciphering the transcriptional landscape of microglia. Through direct sequencing of sorted mouse microglia, Hickman and colleagues revealed signatures that were highly specific for microglia, including *P2ry12, P2ry13, Tmem119, Gpr34, Siglech, Trem2*, and *Cx3cr1*. These components constitute the microglial sensing apparatus, often referred to as the microglial “sensome” [185]. A comparative analysis of microglial and glial transcriptomes revealed that the genes *P2ry12, Fcrls, Tmem119, Olfml3, Hexb, and Tgfbr1* are microglia-specific and that *TGF-β1* is needed for the development of microglia [186].

Previous studies aimed at delineating the transcriptome of human microglia reported enhanced expression of genes implicated in microglial ramification and motility (*P2RY12* and *CX3CR1*), synaptic remodeling (*C3, C1QA, C1QB*, and *C1QC*), and the immune response (*HLA‐DRA* and *HLA‐B*) in autopsy samples from patients with epilepsy, brain tumors or acute ischemia [187, 188]. Moreover, Gene Ontology (GO) analysis of the microglial transcriptome isolated from postmortem brain tissues revealed enrichment of GO terms associated with immune signaling and modulation (*CD74, CSFR1*, and *C1QA‐C*), pathogen and self‐recognition (*MyD88, CLECL1*, and *CIITA*), and cell adhesion and motility (*ITGAM, CX3CR1*, and *ICAM‐1*) [188], underscoring the central role of microglia in the diseased brain.

The classical techniques used to isolate microglia often require lengthy preparation procedures that potentially introduce ex vivo transcriptional artifacts. An alternative strategy to study microglia-specific signatures in vivo is the ribosomal tagging (RiboTag) approach, which relies on Cre recombinase‐induced expression of a hemagglutinin (HA) tag fused to the core ribosomal protein 22 (Rpl22) [189], allowing rapid pulldown of RNA. The RiboTag approach provides a snapshot of RNA actively undergoing translation at a precise time point. By exploiting the RiboTag approach in longitudinal translatomic analyses of microglia in a relapsing–remitting experimental autoimmune encephalomyelitis (EAE) model, Haimon et al. [190] showed the enrichment of microglial genes associated with proliferation (*Birc5, Lig1*, and *Top2a*), antigen processing and presentation (*Ciita, Cd74*, and *H2-Ab1*), and the response to IFN-γ, suggesting the establishment of mutual interactions between disease-experienced microglia and T cells. With a similar strategy, Acharjee et al. [191] observed highly dynamic microglial immune responses in various stages of EAE that differed to some extent between the brain and spinal cord. Collectively, these studies indicate that microglia exhibit regional and functional heterogeneity, and since RNAs from bulk RNA-seq are analyzed *en masse*, such cellular heterogeneity is often diluted or even missed entirely.

### Single-cell RNA sequencing

Since its invention more than a decade ago, single-cell RNA sequencing (scRNA-seq) has been transformative for understanding microglia-mediated functions in various neurodegenerative diseases [192, 193] and viral infections [179, 194, 195]. This technique deconvolutes cell heterogeneity, thus allowing the identification of microglial subsets that are associated with certain microglial phenotypes. Using 5xFAD mice, which constitute an animal model of Alzheimer’s disease (AD), Keren-Shaul et al. [192] identified novel microglial clusters exhibiting distinct expression signatures that were annotated as neurodegenerative disease-associated microglia (DAMs). Compared with homeostatic microglia, DAMs exhibited reduced expression of core microglial genes (*P2ry12, Tmem119, Csf1r*, and *Cx3cr1*) and increased expression of AD risk genes (*Apoe, Lpl, Trem2, Tyrobp*, and *Ctsd*), suggesting that DAMs may directly affect disease progression. In addition, Deczkowska et al. [196] reported DAM signatures in the SOD1-G93A model of amyotrophic lateral sclerosis, highlighting that DAMs represent a general response to different neurodegenerative diseases. In addition to DAMs, Mathys et al. [193] identified two distinct reactive microglial types expressing IFN genes (*Ifitm3, Irf7, OaS1a*, and *Zbp1*) and MHC genes (*H2-D1, H2-Aa, H2-Ab1*, and *Cd74*) in the hippocampus of a cyclin-dependent kinase 5 (CK-p25) mouse model. A trajectory analysis revealed the continued progression of homeostatic microglia to an activated state, which ultimately branches into DAM, IFN-I, and MHC terminal states [197]. Notably, IFN- and MHC-expressing microglia were not unique to neurodegenerative models, as subsequent scRNA-seq studies on viral infection, LPS stimulation, and glioma identified them as well. Syage et al. [198] studied JHMV infection and identified microglial clusters with increased levels of proinflammatory genes (*Ccl3, Ccl4*, and *Cxcl10*) that appeared to be more extensively associated with the disease. Specifically, genes encoding antiviral factors, including *Myd88*, *Rsad2* (Viperin), and *Tmem173* (STING), were highly upregulated in virus-exposed microglia, indicating a central role for the microglial sensome in orchestrating the antiviral defense that aids in controlling JHMV replication. Indeed, ablation of MAVS in the brain was shown to induce defective interferon responses in microglia, dysregulated lymphocyte infiltration and enhanced susceptibility to RVFV [68]. In the WNV-infected brain, Spiteri et al. [199] reported that microglia adopted unique and global transcriptomic profiles in the lethal progression of WNE. Pseudotime projection revealed the transition of microglia from homeostatic to antiviral microglia expressing the genes *Tlr3, Tlr7, Ddx58* (RIG-I), *Myd88*, *Irf7*, *Stat1*, and *Ifitm3* at the early onset of disease and then to immune cell-recruiting microglia expressing an armamentarium of chemotactic signals, including *Ccl3, Ccl4, Ccl5, Cxcl16, Cxcl9*, and *Il-12b*. Indeed, *Il-12b* promotes the differentiation of naïve CD4^+^ T cells into T_H_1 cells [200] in response to T-cell-derived IFN-γ in the CNS [201]. Although T cells are critical for CNS viral control, microglia are pivotal for their recruitment or maintenance within the inflamed CNS. A recent study by Rosen et al. [179] identified putative ligand–receptor pairs involved in intercellular communication between microglia and T cells. Through genetic and pharmacological manipulation, the CXCL16-CXCR6 axis was shown to be vital for the maintenance and differentiation of WNV-specific CD8^+^ TRM cells in the postinfectious CNS [179]. The persistence of T cells after viral clearance contributes to microglial activation [202, 203], leading to synapse elimination in the CA3 region of the hippocampus in WNV-infected animals [179].

### Spatiotemporal omics

While single-cell sequencing platforms provide a higher resolution of microglial profiles, they require the isolation of cells from tissues, which inherently leads to a loss of spatial information. Spatial transcriptomic platforms could validate such a hypothesis by providing a more integrated analysis that correlates microglial activation profiles with a specific microenvironmental niche defined by its cellular composition and tissue architecture. Maniatis and colleagues reported the regional and temporal dynamics of microglia in amyotrophic lateral sclerosis (ALS) using both mouse and human spinal cord tissue [204]. Indeed, they reported increased expression of *Tyrobp* and *Trem2* in the ventral horn and ventral white matter, suggesting that TREM2- and TYROBP-mediated signaling is an early step in disease-relevant changes in microglial gene expression. Choi et al. [205] reported abundant expression of *Ctsd, Tyrobp, C4b, Lyz2, Cst7*, and *Ctsz* in microglia in the cerebral cortex, hippocampus, and striatum in the 5XFAD model. The spatial distribution of the DAM signatures showed an increase in the gray matter of 7-month-old 5XFAD mice, which reflects an AD phenotype, and a relatively prominent increase in the white matter of 3-month-old 5XFAD mice [205], re-emphasizing region- and age-dependent microglial responses to AD. Although microglia display a stereotypically activated response to β-amyloid (Aβ) [192, 206, 207], little is known about the relationship between amyloid plaques and the neurodegenerative process [208–210]. Nevertheless, recent studies revealed the expression of plaque-induced genes (PIGs) in immediate proximity to plaques in an AD mouse model [211, 212]. The expression of PIGs is positively correlated with plaque density in different brain regions and is functionally involved in phagocytic and degradative processes [212]. Gratuze et al. [213] and Kulkarni et al. [214] discuss the function of TREM2 in microglia. In brief, TREM2 deficient microglia show diminished responses upon IL-4 stimulation [215], and TREM2 has been reported to be instrumental in the increased density of microglia around plaques [212]. Considering these findings, the hypothesis that microglial contact with plaques is necessary for microglia to respond appropriately to amyloid pathology appears reasonable.

Spatial transcriptomics of microglia has been hampered by the relatively low resolution, low multiplexing capabilities and very few cells that were analyzed to validate specific genes of interest [216, 217]. Recently, single-cell proteomics, including multiplexed ion beam imaging by time-of-flight (MIBI-TOF) and imaging mass cytometry (IMC), has been developed to capture microglia within the intricate multicellular microenvironments of the brain [218, 219]. By profiling the protein spectrum, Mrdjen et al. [220] identified unique microglial phenotypes summarized as the microglial state continuum (MSC), which progressively expresses HLA-DR, MerTK, CD11c, MRP14, and TREM2 within compartmentalized brain regions. Notably, the hippocampus and substantia nigra (SN) were reported to harbor a high MSC, suggesting that microglia in these regions are skewed toward a more active state. In contrast, under AD conditions, hippocampal microglia dampen MSC states, presumably indicating that microglia from hippocampal gray matter under AD conditions are less activated or more senescent than microglia from healthy hippocampal gray matter. Vijayaragavan et al. [218] found that microglia increasingly express DAM signatures, which interact strongly with pathological Tau in the CA1 region of a patient with AD dementia. By exploiting IMC, Schwabenland et al. [221] highlight the spatial resolution of CNS-related encephalopathies, such as microglial nodules, in COVID-19 patients. Indeed, microglial nodules represent microanatomic immune niches enriched with activated CD8^+^ T cells [221].

## Current insights into viral infections as potential triggers and accelerators of neurodegeneration

Viral infections of the brain can lead to acute and chronic encephalitic processes involving acute and long-term neurological deficits [1]. In all these scenarios, neuronal death and neuronal loss can be hallmarks of the respective conditions [1]. Notably, during viral encephalitis, neurons can undergo apoptosis or cell death in the acute phase, as well as in the chronic phase of infection. However, the mechanisms leading to neuronal loss and death can differ depending on the time course of infection and the composition of the surrounding cells. Nonetheless, neuronal loss often leads to long-term sequelae, cognitive deficits, and motor impairments even months after JEV and WNV infection [6, 222]. Moreover, according to the WHO [223], the risk of developing epilepsy, which is one of the most common chronic neurological diseases among humans that can manifest years after survival of a JEV infection is up to 30%. Autoimmune encephalitis develops in approximately 25% of HSE-recovered patients, often months after the initial recovery [3]. Patients typically develop autoantibodies against neuronal antigens such as the N-methyl-D-aspartic acid (NMDA) receptor in the absence of viral detection in cerebrospinal fluid (CSF), which results in seizures, cognitive dysfunction and choreoathetosis [3].

During acute viral encephalitis, e.g., encephalitis caused by HSV-1, spontaneous symptomatic seizures are often observed in patients. These effects can be triggered by robust inflammatory antiviral responses within brain tissue, mostly accompanied by the secretion of a broad range of proinflammatory cytokines, such as TNF, IL-6, and IL-1β, which have been shown to be involved in acute symptomatic seizure development [186, 224–226]. Infiltrating monocytes, especially microglia, are known to actively produce these cytokines [227]. Notably, acute seizures develop during the acute phase of viral encephalitis, and neuronal loss can be observed at these early stages. In a mouse model of viral encephalitis and temporal lobe epilepsy (TLE), hippocampal neurons undergo cell death within the first six days post virus infection, suggesting a direct neurotoxic effect that is mediated by either the virus itself or by the initiated immune response [228–230]. However, microglial depletion experiments in models of viral CNS infection have shown that microglia are essential for the survival of the host and that the absence of microglia leads to an exacerbation of CNS pathology [27, 98, 99, 101, 102]. Therefore, these resident immune cells are crucial for protection in the acute phase of viral encephalitis. However, microglia are known to mediate synaptic remodeling and synaptic elimination in a mouse model of WNV-induced cognitive dysfunction upon viral clearance via complement secretion and IFN-γ sensing [120, 121]. Complement C3 deficiency can ameliorate synaptic remodeling and reverse spatial learning deficits in mice upon WNV infection [121]. Currently, complement-targeting pharmaceuticals are being tested with promising results in clinical trials for the treatment of severe neurological disorders [231]. Furthermore, the lipid-binding receptor TREM2, which is predominantly expressed by tissue macrophages such as microglia, has been shown to modulate the risk of late-onset AD in genome-wide association studies [232]. A meta-analysis of studies with HSV-1 viral DNA data or seropositivity detection in individuals with AD suggested a correlation between HSV-1 infection and the risk of developing AD [233]. In this regard, TREM2 expression levels are of central relevance for microglial function, and TREM2 was recently shown to be downregulated upon HSV-1 infection in human iPSC-derived microglia [234].

During the COVID-19 pandemic, many patients experienced neurologic complications and even long-term sequelae, including encephalitis/encephalopathies, Guillain–Barré syndrome, stroke, and seizures [235]. Furthermore, irrespective of the severity of acute respiratory disease during SARS-CoV-2 infection, postmortem autopsies of patients who succumbed to COVID-19 revealed only minimal detection of viral copies within the brain, which indicates that the observed neurological sequelae are unlikely to be caused by direct CNS invasion of the virus [236]. However, studies have shown that brain injury markers, such as NFL, GFAP, and tTau, are elevated in the CSF of neuro-COVID-19 patients during the acute and chronic phases [235, 237, 238]. These elevated levels also correlate with increased levels of inflammatory cytokines, such as IL-6, CCL2, IL-1RA, and IL-12p40 [235]. These studies suggest that the late innate immune response signatures of hosts are associated with markers of dendritic and axonal injury, which indicates neuronal loss. Many hypotheses about the exact mechanisms underlying how neuropathology develops in COVID-19 patients have been formulated and investigated in patient cohorts and in vivo studies, including the involvement of IL-6 and different isoforms of cytokines, the complement pathway and microthrombosis, neuronal loss and degeneration, and increases in IL-1β and IL-6 levels within the hippocampus, leading to decreased neurogenesis and memory impairment [235]. However, no correlation with respiratory disease severity or neuropathology was observed in these studies. Due to the usually undetectable levels of the virus in the CNS, the neuropathology seems to be mediated by the immune response, glial activation, and glial metabolism [235, 239, 240].

Viral CNS infections have also been linked to neurodegenerative diseases, such as Parkinson’s disease (PD). A pathophysiological hallmark of PD is the accumulation of aggregated α-synuclein or tau in the brain, which is suspected to lead to neuronal degeneration in distinct brain areas [241]. Symptoms do not manifest until a critical number of neurons are lost, which complicates the development of treatments and the design of clinical studies [241]. Some epidemiological evidence suggests that infections might trigger and/or accelerate neurodegenerative diseases such as PD. The encephalitis lethargica “epidemic” is an example of a parkinsonian symptom that occurred after the Spanish flu pandemic at the beginning of the twentieth century, although the exact pathophysiology of that phenomenon remains unclear [242, 243]. The IAV strain H5N1 can migrate into the CNS and lead to chronic microglial activation and α-synuclein aggregation followed by neuronal loss in the SN of mice [244]. Infection with a neurotropic IAV induced prion protein misfolding, suggesting that viruses can potentially initiate protein aggregation and/or misfolding. Furthermore, evidence for the presence of IAV in the SN of PD patients was obtained postmortem [245]. In a mouse model of viral encephalitis caused by western equine encephalitis virus (WEEV), persistent activation of microglia and astrocytes was observed, which led to the aggregation of α-Syn and subsequent loss of dopaminergic neurons in the SN [246]. A large meta-analysis showed that patients with a history of infections, especially bacterial infections, had a 20% greater risk of developing PD (OR 1.20) [247]. Similarly, a prospective cohort study from Germany revealed that gastrointestinal infections increase the risk of developing PD [248]. Nonetheless, the exact mechanisms by which infections enhance the risk of neurodegenerative diseases such as PD and AD remain elusive and are currently under investigation. However, the exploration of antiviral treatment options and identification of new treatment targets are essential for overcoming neurological long-term sequelae in patients suffering from viral CNS infections.

## Summary and outlook

During the last few years, a rather detailed view of the mechanisms of virus control within the infected CNS has emerged. Many observations made in patients with viral encephalitis could be further validated in cell culture and mouse models. However, the key elements involved in virus control within the infected CNS are still not understood. The latest technological developments will certainly help researchers obtain a more detailed understanding of the spatiotemporal conditions of virus control within the CNS. In the future, the focus will be on improving the understanding of cell‒cell interactions between brain-resident cells and -infiltrating cells. Furthermore, deciphering the relevant mechanisms of local restimulation of brain-infiltrating antigen-specific T cells and how their function adapts to local requirements will be important. Recently exploited adoptive T-cell therapies for the treatment of progressive multifocal leukoencephalopathy (PML) [249] provide hope that new treatments can be developed, based on a better understanding of the local conditions of viral encephalitis.
